# Physical Therapists’ Choices, Views and Agreements Regarding Non-Pharmacological and Non-Surgical Interventions for Knee Osteoarthritis Patients: A Mixed-Methods Study

**DOI:** 10.31138/mjr.34.2.188

**Published:** 2023-06-30

**Authors:** Ricardo M. Ferreira, Pedro N Martins, Nuno Pimenta, Rui S. Gonçalves

**Affiliations:** 1 Physical Exercise and Sports Department, Polytechnic Institute of Maia, N2i, Maia, Portugal,; 2 Physical Therapy Department, Coimbra Health School, Polytechnic Institute of Coimbra, São Martinho do Bispo, Coimbra, Portugal,; 3 Physical Exercise and Sports Department, Polytechnic Institute of Bragança, Bragança, Portugal

**Keywords:** physical therapy, knee osteoarthritis, non-pharmacological interventions, non-surgical interventions

## Abstract

**Objective/Aims:**

The aims of this study are to collect the most common non-pharmacological and non-surgical interventions used by the Portuguese physical therapists in their knee osteoarthritis patients, and to deeper understand the factors associated to their intervention choices.

**Methods:**

This study incorporated a mixed-methods design. For the quantitative data it was choose an e-survey (with 25 close-end questions, plus general information of the study and a clinical vignette), retrieving sociodemographic and self-reported practice on knee osteoarthritis information. It was analysed response frequencies and associations between variables with logistic regression analyses. For the qualitative data, it was chosen to perform semi-structured interviews in purposefully selected physical therapists to include different sociodemographic factors and survey responses regarding the physical therapists’ interventions chosen. After the interviews, the audios were collected, anonymised, transcribed verbatim, and the texts explored by the thematic approach.

**Results:**

From the 277 PTs that shown interest in participating in the study, 120 fully completed the questionnaire and, from those, 10 participated in the interviews. The most chosen interventions included Resistance Exercise, Manual Therapy, Nutrition/Weight Loss, Self-care/Education, Stretching and Aquatic Exercise. Furthermore, it seems that PTs’ individual characteristics (age, experience, and clinical reasoning), patient’s characteristics (clinical findings and preferences), and work-related factors (facility type, work environment and available resources) are the main actors responsible for an intervention chosen.

**Conclusions:**

In the Portuguese PTs context the most important interventions are Exercise, Manual Therapy, Nutrition/Weight Loss, and Self-care/Education; these interventions chosen may be influenced by PT, patient and work-related factors.

## INTRODUCTION

Osteoarthritis (OA) is the most common form of arthritis and, from all joints, the knee OA is the most prevalent.^1,2^ Current knee OA rehabilitation strategy is a complex process, where it may be used surgical and non-surgical interventions.^3–5^ There are several non-pharmacological and non-surgical interventions that can be used to manage patients with knee OA, the majority physical therapy related.^6–10^ Despite being widely used to manage patients with knee OA, physical therapy practice has been subjected to decades of criticism for its lack of research, and is often perceived as a profession that bases its practice largely on anecdotal evidence, using treatment techniques that have little scientific support.^11^ This was identified, as early as 1969, to be a significant issue for the physical therapy profession.^12^ Over the years, many efforts were made to increase physical therapy research^13^ and to shift from the traditional models of practice (guided on the therapist tacit knowledge and opinion) to a more evidence-based practice (EBP) overtime.^11,14,15^

So, the aims of this study are to collect the most common non-pharmacological and non-surgical interventions used by the Portuguese physical therapists (PTs) in their patients with knee OA, and deeper understand the factors associated to their intervention choices.

## MATERIAL AND METHODS

This study incorporated a concurrent mixed-methods design^16–19^ and followed the Ethical Principles of the Helsinki Declaration (2013).^20^ Additionally, it was approved by the ethics committee (CEFADE24-2019) and all PTs enrolled were informed and signed the individual inform consent form.

### Sample

In an attempt to ensure the correct population sample, the national physical therapy professional association (APFISIO) e-mail database was requested for the Portuguese PTs working class recruitment. Also, in order to increase the number of enrolled participants, the e-mails of past students from all physical therapy national schools were requested.

### Design – Quantitative

For the quantitative data, it was chosen to apply a self-administered e-survey. The e-survey was evaluated, designed, administered, conducted and collected according to established guidelines.^21–23^

The e-survey was initially e-mailed all voluntary PTs in the APFISIO database in the regular online newsletter and to past PT students as a formal e-mail with a cover letter containing the study’s information (background, justification and aims). Additionally, after reading the study’s information, the participants were invited to click in the e-survey link (https://pt.surveymonkey.com/r/PBE2019FADEUP). When clicking the link, the participants were then connected to the SurveyMonkey and forwarded to the e-survey. Before initiating the e-survey, the informed consent, the data protection rights, and how the results will be used (analysed anonymously and confidentially, the data gathered was only used for statistical information in an academic environment), the criteria for selecting the participants and the reasons for non-participation, the possibility to stop the e-survey at any time, the information that no incentives will be provided, instructions how to fill and complete the e-survey, and e-mail address for possible clarifications, were explicitly stated. The e-survey included 25 close-ended questions, divided into 2 main stages (the e-survey may be found in the supplemental data):

1. Sociodemographic information. At this stage, in addition to collecting sociodemographic information, the participants’ eligibility was also analysed with the inclusion and exclusion criteria:
  - Inclusion: have an active physical therapy license; obtained at least the physical therapy bachelor’s degree; work or have worked as a PT in the past 6 months in Portugal; be able to read, write, and speak Portuguese.
  - Exclusion: do not have an active physical therapy license or have another profession than PT; obtained the physical therapy bachelor’s degree in a foreign country; does not work in Portugal; is not be able to read, write or speak Portuguese; be a physical therapy bachelor student.
  - Most frequently used non-pharmacological and non-surgical interventions applied in patients with knee OA. The respondents were invited to rank by preference 5 non-pharmacological and non-surgical interventions for managing patients with knee OA, from 31 available interventions options. The interventions options were achieved after a preliminary literature search. In order not to bias the PTs interventions choices, the interventions appeared in a random order, not repeating its order from e-survey to e-survey. For helping to contextualise, a knee OA clinical vignette was provided (translated to Portuguese from the Holden et al.^24^ study).

Before sending the e-survey by e-mail, the e-survey was pre-tested by the authors and evaluated in its completion time, design, questions order, attractiveness, syntax, clarity, logic, correct question types, and response format. Also, it was permitted to the respondents to review and change their answers. The sample size goal for this study was 373 responses, based in a 95% confidence level, a margin of error of 5% and a 50% response distribution.^25^ To ensure that the sample size goal was achieved, after two, four and six weeks respectively, a thank you note and a reminder containing the e-survey link was e-mailed. In an attempt to avoid duplication filled questionnaires, only responses were accepted for each IP address.

### Design – Qualitative

For the qualitative data collecting, it was chosen to apply semi-structured interviews with open-ended questions on the PTs. The interviews were conducted by 1 PhD and methodological experienced author, blinded to the PTs characteristics and prior questionnaire answers, using Skype (Microsoft Corporation, Rives de Clausen, Luxemburg). Only audio-recorded was performed – excluding any face-to-face or written contact. There was no relationship between the interviewer and the PTs prior to the study, and the interviewees were blind regarding the interviewer’ characteristics (an “anonymous” e-mail and Skype account were created). The interviewees were recruited by completing the study during previous stages where, following a review of questionnaire responses, the sample was purposefully selected to include different sociodemographic factors and interventions responses for patients with knee OA. To ensure a high participation rate, after one, two, and four weeks respectively, a thank you note, a reminder containing the interview objectives, and a request to provide their most convenient dates/times for the interview, were e-mailed. The semi-structured interviews were performed according to Leech et al.^26^ guidelines. The questions in the interview script were constructed according to Qu et al.^27^ The interview script was properly validated by an external expert panel (of 2 independent and methodological experienced PhDs), where there were able to comment and suggest improvements. Before initiating the “core” questions, an introductory section with the purpose of the study, the protection rights, how the data will be used and some warm-up questions were included in order to build empathy and comfort. The “yes” or “no” answers were avoided. At the end of the core questions, it was given the opportunity for the interviewees to add information and opinions that they found to be relevant. Additionally, the interview script was tested on the first participant who, after the interview, was asked for feedback on the interview conduction, structure, design and phrasing of questions. The script may be consulted in the supplemental data.

### Data Analysis – Quantitative

Response frequencies were analysed using Microsoft Excel and IBM SPSS 26.0 software. After examining the response frequencies, the variables categories were collapsed. In the interventions choices, the “1^st^”, “2^nd^”, “3^rd^”, “4^th^”, and “5^th^” were combined so that a 2-category response was obtained: “Present” (if the PT chooses 1^st^, 2^nd^, 3^rd^, 4^th^ or 5^th^) or “Absent” (no intervention choice). Additionally, in sociodemographic data where subsamples were smaller, we collapsed categories in an effort to derive stable models. The Certificate and Baccalaureate degrees into the same category (Baccalaureate) – as in Portugal they are the minimum required professional entry-level – and our sample included only 1 PT who indicated a professional Post-Doctorate degree, so we included him/her with others PhD degrees. After item categories were collapsed, logistic regression analyses were conducted to examine the associations with the PTs’ characteristics. An alpha level of 0.05 was used to determine whether a model was to be reported. Odds ratios (OR) and their 95% confidence intervals (CIs) were determined for each level of the independent variables in those models that were significant.^28^

### Data Analysis – Qualitative

The data was analysed with a Computer Assisted Qualitative Data Analysis Software, namely the NVivo v12 (QRS International, Doncaster, Victoria, Australia).^29^ The audios collected in the interviews were anonymized and verbatim transcribed. Then the texts were explored by 3 authors with the thematic approach.^30^ The original classification tree was analysed and further discussed with an external expert panel of 3 methodological experienced PhDs,, where some categories were collapsed, eliminated, or renamed. Quotations were identified to report the findings and illustrate the content, and were translated from Portuguese to English. To ensure complete and transparent data reporting, the methodology was conducted according to established guidelines.^31–35^

## RESULTS

### Quantitative

**Figure 1.**
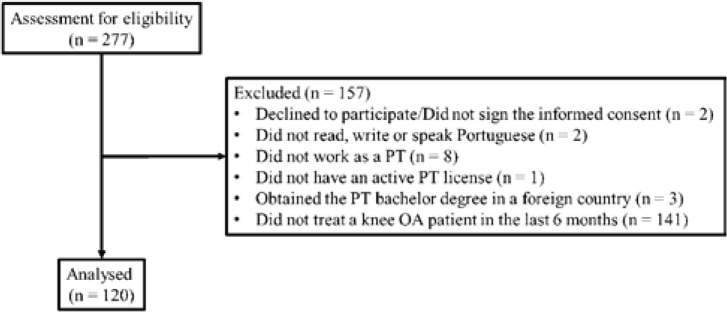
Questionnaire Participation and Completion.

From the 227 PTs that shown interest in participating in the study, only 120 (52.9%) fully completed the questionnaire (**Figure 1**). The descriptive statistics of the PTs personal and practice characteristics are presented in **Table 1**.

**Table 1.** PTs’ Personal and Practice characteristics.

**Characteristic**	**Frequency (%)**
*Sex*	
Male	36 (30%)
Female	84 (70%)
*Age Groups*	
20–29 years	34 (28.3%)
30–39 years	54 (45%)
40–49 years	13 (10.8%)
≥ 50 years	19 (15.8%)
*Valid License*	
< 5 years	18 (15%)
5–10 years	41 (34.2%)
11–15 years	30 (25%)
> 15 years	31 (25.8%)
*Degree*	
Certificate	3 (2.5%)
Baccalaureate	81 (67.5%)
Master	30 (25%)
Doctorate	5 (4.2%)
Post-doctorate	1 (0.8%)
*Pursue a Higher Academic Degree*	
Yes	80 (66.7%)
No	16 (13.3%)
Do Not Know	24 (20%)
*Participate in Continuing Education*	
Yes	172 (89.1%)
No	21 (10.9%)
*Belong to a Practice-oriented Organization*	
Yes	107 (89.2%)
No	13 (10.8%)
*Instructor*	
Yes	59 (49.2%)
No	61 (50.8%)
*Certificate/Baccalaureate School*	
ESSATLA	9 (7.5%)
ESSCVP	4 (3.3%)
ESSUA	1 (0.8%)
ESSL	3 (2.5%)
ESSP	8 (6.7%)
*Working Hours per Week*	
20–30	18 (15%)
31–40	44 (36.7%)
> 40	58 (48.3%)
*Patients per Day*	
1–5	13 (10.8%)
6–10	39 (32.5%)
11–15	30 (25%)
> 15	38 (31.7%)
*Number of PTs in the Facility*	
0	23 (19.2%)
1–5	56 (46.7%)
6–10	21 (17.5%)
11–15	9 (7.5%)
> 15	11 (9.2%)
*Percentage of Total Work Time in:*	
Patient Care	
5–25%	5 (4.2%)
30–50%	12 (10%)
55–75 %	32 (26.7%)
80–100 %	71 (59.2%)
Researcher	
0%	55 (45.8%)
5–25%	53 (44.2%)
30–50%	10 (8.3%)
55–75%	2 (1.7%)
Teacher	
0%	74 (61.7%)
5–25%	28 (23.3%)
30–50%	9 (7.5%)
55–75 %	6 (5%)
80–100 %	3 (2.5%)
*Location of the Facility*	
Rural	8 (6.7%)
Suburban	18 (15%)
Urban	94 (78.3%)
*Facility District*	
Açores	4 (3.3%)
ESSS	9 (7.5%)
ESSA	25 (20.8%)
ESSVA	4 (3.3%)
ESSVS	5 (4.2%)
ESSLD	9 (7.5%)
ESSEM	4 (3.3%)
ESSJP – Vila Nova de Gaia	4 (3.3%)
ESSJP – Viseu	1 (0.8%)
ESTeSC	18 (15%)
ESTeSL	8 (6.7%)
ISSAA	3 (2.5%)
UFP	5 (4.2%)
Aveiro	9 (7.5%)
Braga	5 (4.2%)
Bragança	2 (1.7%)
Castelo Branco	2 (1.7%)
Coimbra	9 (7.5%)
Faro	1 (0.8%)
Guarda	3 (2.5%)
Leiria	7 (5.8%)
Lisboa	44 (36.7%)
Madeira	3 (2.5%)
Portalegre	1 (0.8%)
Porto	14 (11.7%)
Santarém	2 (1.7%)
Setúbal	8 (6.7%)
Viana do Castelo	3 (2.5%)
Vila Real	1 (0.8%)
Viseu	2 (1.7%)
*Type of Facility*	
Town Hall	1 (0.8%)
Physical Medicine and Rehabilitation	21 (17.5%)
Center	
Health Center	3 (2.5%)
Geriatric Center/Resting Home	15 (12.5%)
Private Clinic	28 (23.3%)
Sports Club	1 (0.8%)
Home Care	2 (1.7%)
Physiotherapy Office	13 (10.8%)
Private Hospital	3 (2.5%)
Public or Public-Private Partnership Hospital	20 (16.7%)
Continuing Care Unit	13 (10.8%)
*Majority of Patients Condition*	
Cardiovascular/pulmonary	5 (4.2%)
Palliative Care	8 (6.7%)
Hospital Health Care	4 (3.3%)
Sport	4 (3.3%)
Aging	19 (15.8%)
Aquatic Physiotherapy	2 (1.7%)
Orthopedic	62 (51.7%)
Neurological	12 (10%)
Paediatric	2 (1.7%)
Women’s Health	1 (0.8%)
Other	1 (0.5%)
*Majority of Patients Age Group*	
Paediatric (≤ 18 years)	2 (1.7%)
Adult (19–64 years)	75 (62.5%)
Geriatric (≥ 65 years)	43 (35.8%)
*Work Sector*	
Public	33 (27.5%)
Private	80 (66.7%)
Academic	7 (5.8%)
*Work Modality*	
Own Account	30 (25%)
Someone Else’s Account	90 (75%)

ESSATLA: Escola Superior de Saúde Atlântica; ESSCVP: Escola Superior de Saúde da Cruz Vermelha Portuguesa; ESSUA: Escola Superior de Saúde da Universidade de Aveiro; ESSL: Escola Superior de Saúde de Leiria; ESSP: Escola Superior de Saúde do Porto; ESSS: Escola Superior de Saúde de Setúbal; ESSA: Escola Superior de Saúde de Alcoitão; ESSVA: Escola Superior de Saúde do Vale do Ave; ESSVS - Escola Superior de Saúde do Vale do Sousa; ESSLD: Escola Superior de Saúde Dr. Lopes Dias; ESSEM: Escola Superior de Saúde Egas Moniz; ESSJP: Escola Superior de Saúde Jean Piaget; ESTeSC: Escola Superior de Tecnologia e da Saúde de Coimbra; ESTeSL: Escola Superior de Tecnologia e da Saúde de Lisboa; ISSAA: Instituto Superior da Saúde do Alto Ave; UFP: Universidade Fernando Pessoa.

**Figure 2.**
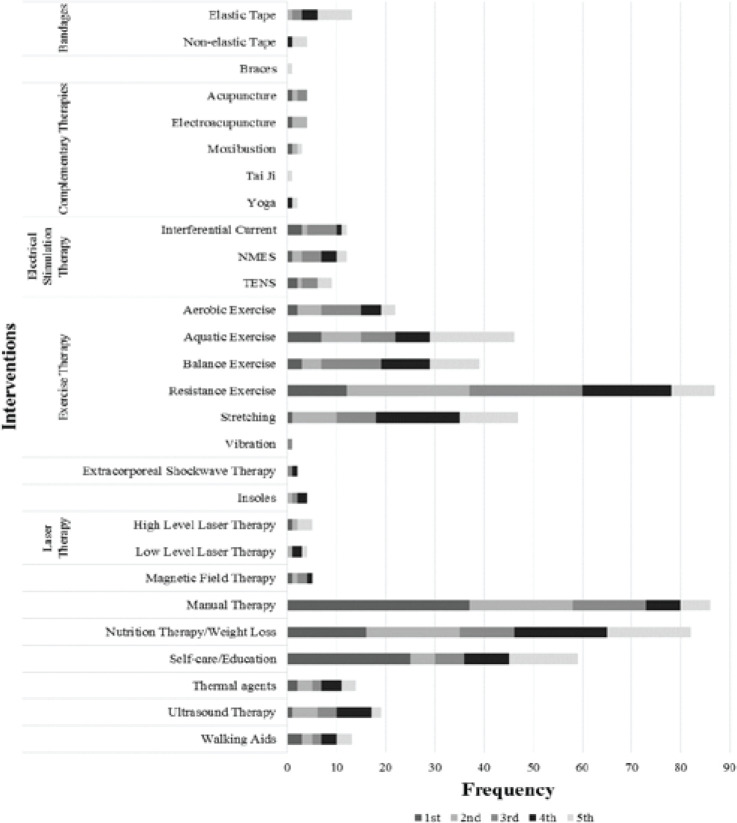
PT’s Interventions Choices. NMES: Neuromuscular Electrical Stimulation; TENS: Transcutaneous Electrical Nerve Stimulation. Note: As Balneotherapy/Spa, Cupping Therapy and Leech Therapy interventions were not chosen by the PTs, they are not displayed.

The six most chosen interventions were Resistance Exercise (14.5%), Manual Therapy (14.3%), Nutrition/Weight Loss (13.7%), Self-care/Education (9.8%), Stretching (7.8%) and Aquatic Exercise (7.7%). The interventions medium chosen were Elastic Tape, Electrical Stimulation Therapies (Interferential Current [IFC], Neuromuscular Electrical Stimulation [NMES] and Transcutaneous Electrical Nerve Stimulation [TENS]), Aerobic Exercise, Balance Exercise, Thermal Agents, Ultrasound Therapy (US) and Walking Aids – all between 6.5 and 1.5%. The least chosen interventions were Non-elastic Tape, Braces, Complementary Therapies (Acupuncture, Electroacupuncture, Moxibustion, Tai Ji and Yoga), Vibration, Extracorporeal Shockwave Therapy, Insoles, Laser Therapy (High Level and Low Level), Magnetic Field Thera py – all below 1% – highlighting the Balneotherapy/Spa, Cupping Therapy and Leech Therapy interventions, as they were not chosen by any PT (0%). Regarding the interventions raking, Manual Therapy was the most chosen for 1^st^ (30.8%), Resistance Exercise for 2^nd^ and 3^rd^ (20.8 and 19.2%, respectively), Nutrition Therapy/Weight Loss for 4^th^ (15.8%) and tied with Aquatic Exercise for 5^th^ (both with 14.2%). The descriptive statistics of the PTs’ interventions choices are presented in **Figure 2**. Also, for a more detailed information, the interventions choices are in the supplemental data.

Additionally, from the 93 intervention combinations found, the two most commonly used were: Balance Exercise + Manual Therapy + Nutrition/Weight Loss + Resistance Exercise + Self-care/Education; and Manual Therapy + Nutrition/Weight Loss + Resistance Exercise + Self-care/Education + Stretching (both combinations with 4.2%). Given the high heterogeneity of interventions used across different combinations, 67.5% were chosen only once (<1%) (**Table 2**).

**Table 2.** Frequency of the combined use of different interventions for treating knee OA patients.

**Aerobic Exercise**	**Aquatic Exercise**	**Balance Exercise**	**Manual Therapy**	**Nutrition/Weight Loss**	**Resistance Exercise**	**Self-care/Education**	**Stretching**	**Ultrasound Therapy**	**N (%)**
		•	•	•	•	•			5 (4.2%)
			•	•	•	•	•		5 (4.2%)
•			•	•	•	•			4 (3.3%)
	•		•	•	•	•			4 (3.3%)
	•		•	•	•			•	4 (3.3%)
•		•		•	•	•			3 (2.5%)
	•			•	•		•	•	3 (2.5%)
		•	•	•	•		•		3 (2.5%)
•	•			•	•	•			2 (1.7%)
•				•	•	•	•		2 (1.7%)
	•	•	•		•		•		2 (1.7%)
		•	•		•	•	•		2 (1.7%)

**Note:** Most interventions combinations (67.5%) were used by <1% of physical therapists and are not displayed.

**Figure 3.**
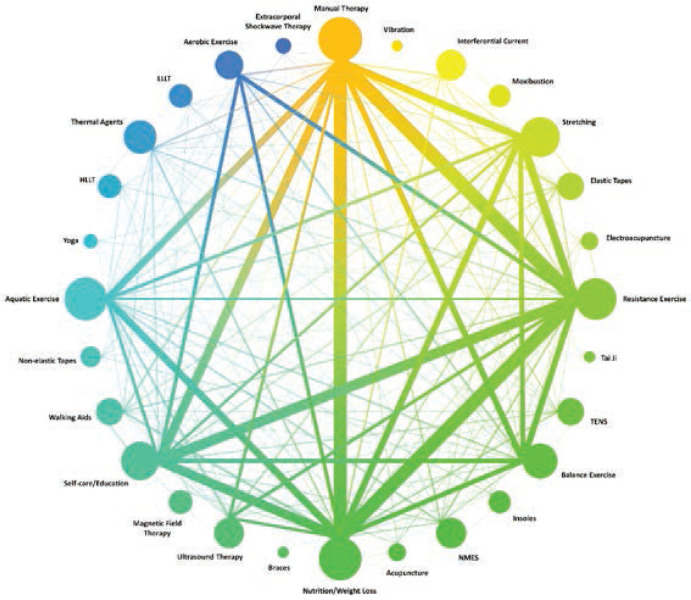
Interventions interactions. The line represents an interaction between two interventions. Its thickness is proportional to the interventions pairs frequency. The dot represents an intervention. Its size is proportional to the number of interventions links. HLLT: High Level Laser Therapy; LLLT: Low Level Laser Therapy; NMES: Neuromuscular Electrical Stimulation; TENS: Transcutaneous Electrical Nerve Stimulation. In interventions, pairs representing less than 1%, transparency was applied in the line.

From the 1200 interventions relations, the two interventions more strongly linked were Manual Therapy + Resistance Exercise (n=62; 5.2%), followed by Nutrition/Weight Loss + Resistance Exercise (n=59; 4.9%), Manu al Therapy + Nutrition/Weight Loss (n=57; 4.8%), Resistance Exercise + Self-care/Education (n=46; 3.8%), Nutrition/Weight Loss + Self-care/Education (n=41; 3.4%), and Manual Therapy + Self-care/Education (n=40; 3.3%). In a note, 75 interventions relations were only found once. From the 28 interventions, the interventions more associated to others were Manual Therapy (n=25; 6.4%), followed by Nutrition/Weight Loss and Aquatic Exercise (n=24; 6.2%), Resistance Exercise (n=23; 5.9%), Stretching (n=22; 5.6%), and Self-care/Education (n=21; 5.4%). **Figure 3** summarises and illustrates the intervention interactions.

Regarding the statistically significant associations between PTs’ characteristics and the most used knee OA interventions, Aerobic and Resistance Exercises were more likely to be chosen by the least experienced therapists, in comparison to more experienced PTs (OR 30.000 [95% CI: 3.337; 269.716] and OR 7.500 [95% CI: 1.469; 38.280]). Still in the Resistance Exercise intervention, the moderate experienced PTs (5–10 years) were 3.9 times more likely to choose it, in comparison to the most experienced therapists. The same pattern was found within the PTs age, where Resistance Exercise was less likely to be chosen by elderly therapists in comparison to their younger peers (20–29 years – OR 9.943 [95% CI: 2.629; 37.605]; 30–39 years – OR 4.898 [95% CI: 1.609; 14.911]; and 40–49 years – OR 9.429 [95% CI: 1.603; 55.447]). Additionally, Self-care was 3.1 times more likely to be chosen by PTs that belong to a professional practice-orientated organization, in comparison to those who do not belong to it. In contrast, PTs that participated in continuing education courses were 74% less likely to choose Balance Exercise, in comparison to other PTs that did not participate in continuing education courses. Additionally, Thermal agents were less likely to be chosen by baccalaureate and PTs that worked in a private sector, comparatively to their academic and doctorate peers (OR 0.077 [95% CI: 0.013; 0.467] and OR 0.108 [95% CI: 0.020; 0.599], respectively). Table III gathers a more detailed information.

**Table 3.** Association between PTs’ characteristics and frequent use of knee OA interventions.

**Interventions (Present)**	**Factor - Level**	**Odds Ratio (95% CI)**	***P***	***R* ^2a^**
Aerobic Exercise				
	*Years of License*		*0.005*	*0.208*
	< 5	30.000 [3.337; 269.716]	0.002	
	5–10	5.143 [0.586; 45.153]	0.140	
	11–15	7.500 [0.844; 66.613]	0.071	
	> 15	Reference^b^		
Balance Exercise				
	*Participated in Continuing Education Courses*		*0.025*	*0.059*
	Yes	0.255 [0.077; 0.840]		
	No	Reference^b^		
Resistance Exercise				
	*Age*		*0.003*	*0.168*
	20–29	9.943 [2.629; 37.605]	0.001	
	30–39	4.898 [1.609; 14.911]	0.005	
	40–49	9.429 [1.603; 55.447]	0.013	
	≥ 50	Reference^b^		
	*Years of License*		*0.022*	*0.120*
	< 5	7.500 [1.469; 38.280]	0.015	
	5–10	3.867 [1.360; 11.000]	0.011	
	11–15	2.578 [0.885; 7.538]	0.084	
	> 15	Reference^b^		
Self-care/Education				
	*Belong to a Professional Practice-orientated Organization*		*0.028*	*0.058*
	Yes	3.141 [1.134; 8.700]		
	No	Reference^b^		
Thermal Agents				
	*Academic Degree*		*0.016*	*0.124*
	Baccalaureate	0.077 [0.013; 0.467]	0.005	
	Master	0.200 [0.031; 1.293]	0.091	
	Doctorate	Reference^b^		
	*Work Sector*		*0.036*	*0.098*
	Public	0.238 [0.040; 1.403]	0.113	
	Private	0.108 [0.020; 0.599]	0.011	
	Academic	Reference^b^		

a Nagelkerke R^2^;

b In logistic regression, one level of the independent variable serve as reference against which the odds of the other levels occurring are determined.

### Qualitative

From the 120 PTs that completed the e-survey only 67 (55.8%) volunteered for the interviews. From those, only 10 responded to the emails. The PTs’ individual characteristics is explored in the supplemental data.

The interviews went from January to April. In the end, 147 minutes of recordings were obtained (15 average – 4 minimum [FT 2]; 22 maximum [FT 8]), which generated 34 transcript pages (3 average – 1 minimum; 5 maximum). The interviews offered compelling fragments of PTs’ experiences about knee OA management. In most cases, the qualitative data underpins the survey findings. The word most often spoken by PTs was persons, followed by pain and techniques (79 times, 58 times and 32 times, respectively). For a more detailed information, consult the word cloud provided in the supplemental data.

With the interviews, the main themes identified were: Interventions (applied, eventually applied, and not applied); Intervention plan rationale; Physical therapy sessions frequency; and Principal and secondary knee OA symptoms. For a more detailed information, the classification tree and codes are in the supplemental data.

**Figure 4.**
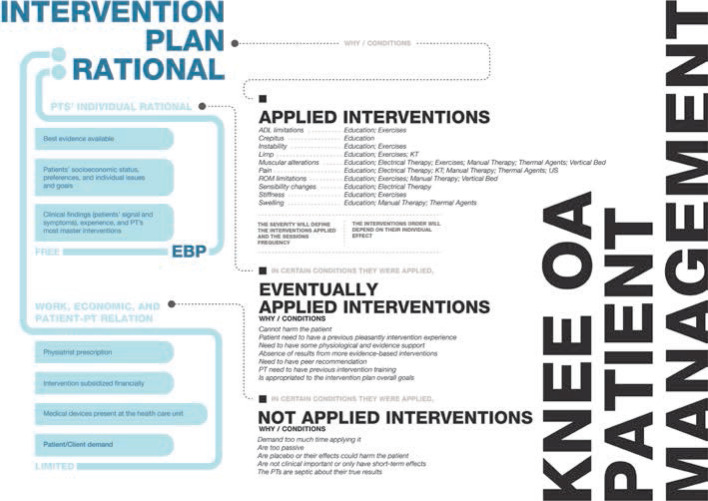
Qualitative data summary and conceptual framework of factors that influence knee OA patient management. ADL: Activities of Daily Living; US: Ultrasound Therapy; KT: Kinesio Tape.

The summary of the qualitative results is described in **Figure 4**. More detailed information is included in the supplemental file texts and quotations.

## DISCUSSION

In the present Portuguese context and after all the data gathering, the most important interventions to manage patients with knee OA are: Exercise, Manual Therapy, Nutrition/Weight Loss, and Self-care/Education.

### Exercise

In the Exercise group, the most important interventions were: Aquatic exercises, Balance exercises, Resistance exercises, and Stretching.

From those, more emphasis needs to be given to Resistance exercises. Resistance exercises was the most chosen intervention in Exercise group (73%), being in 2^nd^ and 3^rd^ place in the general level of importance. This was also found in other countries.^24,36^ The PTs used it mainly because in knee OA, it is expected that patients lose strength progressively.^37,38^ This strength loss may influence not only pain level, but also the patients’ bio-mechanics, function, range of motion (ROM) limitations, quality of life, and activities of daily living.^38–45^ This intervention is highly recommended by evidence for this population (A), being referred in several studies as a “core intervention” for most of the clinical outcomes.^37,39,41,42,44–55^ The second most important intervention in the Exercise group was Stretching (39%). A similar importance was already reported by United Kingdom (UK) PTs.^24^ Stretching exercises are generally associated in the literature with Resistance exercises, as these interventions combined show more clinical importance than Stretching alone.^37,51^ Based in the PTs clinical experience, Stretching may help to relieve muscular tension, and maintain knee ROM and function. As with Stretching, Balance exercises were important to the Portuguese (33%) and UK^24^ PTs. However, their use should also be integrated in the Resistance exercises and individualised according to the patient clinical evaluation.^37,41,46–49^ So, when a patient has muscular weakness and proprioceptive deficits (which can alter balance and postural control), Balance exercises should be integrated in the intervention plan.^56^ This proper patient clinical evaluation importance was further shown in the qualitative and quantitative data, as Balance and Stretching were the only differences in the two most common interventions plans used.

Despite Resistance exercises are easy to perform, safe, effective and do not increase the OA progression,^57^ the PTs explored other exercises options. One of the most referred was Aquatic exercises (18%), scoring 5^th^ place in the general importance level. UK and United States of America PTs also found it important to incorporate them in knee OA patients’ management.^6,24,58^ Although evidence supports it, Aquatic exercises use can be conditioned.^41,42,45 –47,49, 50,59^ Despite often encompassing aspects of aerobic fitness exercises and exercises for enhancing joint ROM, in a low-impact environment,^47,49,60^ the reasons for this conditioning are:^46^ accessibility issues; risk of accidental injury (fall or skin problems); financial burden; poverty of patients; or PTs adhesion. But, as in recent systematic reviews, non-differences were found between land-based and Aquatic exercises, which could be a good alternative for more “fragile” and reluctant patients.^59,60^

Although all patients should be advised to perform exercises to improve both physical and psychological outcomes, they should be patient appropriate.^41,42,54,61–63^ As confirmed in the qualitative data, the exercise movements should be pain free and should respond to the patients’ preferences and clinical findings. So, firstly, preliminary pain relief interventions can be used to allow pain free exercises practice, secondly, patients’ exercises preferences and pain tolerance should be addressed and, thirdly, the exercises should be adapted and adjusted to their individual physical, physiological, social, and emotional characteristics, kinesiophobia, co-morbidities, and other clinical findings. All this will ensure a better exercise plan adhesion and participation.^39,47,54,61–65^

### Manual Therapy

Although a substantial decrease in its use was found compared to previous Portuguese studies,^66–67^ Manual Therapy was still the most important intervention for the Portuguese PTs. In fact, 31% of the PTs chose it in 1^st^ place. This importance may be explained by: (1) Therapy related factors; and (2) Profession related factors. Manual Therapy is a very versatile intervention, has a highly interventions variation, and could be easily combined with other interventions in the PT daily practice (for example, in our study, 25 interactions were found in a sample of 28 interventions).^10,68^ As referred by the PTs (and confirmed in the literature^69^), the most used interventions in this group were massage, passive mobilisations, and soft tissue mobilisation/manipulation techniques. Although different clinical results are expected according to the chosen intervention, their applications were conditioned by the patients’ signs and symptoms, clinical findings and other co-morbidities.^70^ For the Portuguese PT context, the overall objectives were soft tissue relaxation, drainage, pain decrease, and ROM improvement. Additionally, Manual Therapy interventions are generally economic and secure,^10,45,47,71–75^ and the patients, even if the Manual Therapy is considered placebo, feel more comfortable and more enthusiastic if there is a direct contact between PT-patient.^10,75,76^ Therefore, psychological and human behaviour influences may play a role in this intervention.^70,77,78^ Even, as referred by the PTs in the qualitative data, some patients prefer to have an all-passive intervention plan, as active interventions could demand too much physical effort. Unfortunately, this is found in other countries where the patients seek for an ultimate “cure”.^79^

In addition to the factors explained, Manual Therapy is one of the oldest interventions, and has been used all over the world since ancient times.^71^ For many years, physical therapy treatments were based (almost exclusively) in Manual Therapy, distinguishing it from other health professions.^68,75,76,78,80^ Moreover, the hand/fingers/palpation is still widely to access soft tissues texture abnormalities or musculoskeletal dysfunctions, and feeling thickness, swelling, or tightness^75,76,78^; and, touch can be used as a non-verbal communication in the PT-patient interaction.^75^ Although it was not possible to know it in the Portuguese context, in several physical therapies schools worldwide, Manual Therapy still plays an important role in academic curriculums.^76,78,80^ For example, in a study with English and Australian PTs it was found that the main reason for using massage is due to initial training.^81^ Furthermore, in Portugal, the physical therapy symbol is a hand, so it could unintentionally influence the PTs to use it more. This may thus be a cycle that would be hard to break.

Despite being widely used by Portuguese PTs, there is still evidence of non-agreement in its use as some conditionally recommend it^10,37,39,48,50^ and others do not recommend or recommend against.^43,45–47^ Although there was found evidence in pain reduction, and physical performance and function improvements in patients with knee OA,^69–71,82^ the main reasons for this uncertainty are:^70,78,83^ lack of expertise of the healthcare professional (knowledge and skills); there is no complete evidence-based support; difficulty in blind treatment providers and study participants; more than one treatment provider in the studies; and heterogeneity in the studies’ intervention application (technique, force, amplitude, rate, repetition and duration). Other explanations may include:^84^ natural history of disease; regression to the mean; placebo effect; and patient usual behaviour change (Hawthorne effect). Even in our PT sample, there is no agreement in its use, as some PTs thought that Manual Therapies techniques can be too passive and do not provide the desired effects. So, it is recommended whenever possible to combine Manual Therapy with Exercise and avoid an exclusively Manual Therapy isolated intervention plan.^37,47,48^ This was further confirmed in the quantitative data where the two most strongly linked interventions were Manual Therapy and Resistance Exercise. More studies are required to clarify the importance of Manual Therapy in the knee OA management.

### Nutrition/Weight Loss

Although not often associated to the physical therapy profession, Nutrition/Weight Loss was considered important by the PTs (14%). This choice may be a response to a common knee OA patient characteristic, obesity.^39,49^ Weight loss can be achieved by nutritional monitoring and/or exercises.^41,42,49,85,86^ Despite nutritionists being professional, a lack of PTs’ confidence in providing specific orientations may arise, but PTs may also help in the nutritional monitoring by educating the patients with knee OA in living a healthy lifestyle and changing some unhealthy alimentary habits.^72^ Nevertheless, as exercises are more PT profession related, many PTs feel more comfortable to mention and advise exercise than weight lose through nutrition or diet.^87^ Besides Resistance exercises, PTs could also use Aerobic and/or Aquatic exercises. This is all considered to be highly supported by evidence (A)^39,41,46,47,50,53,55,88^ and, as showed in the qualitative data, their choice will depend on: patients’ preferences; adhesion and individual characteristics; clinical findings and patients’ co-morbidities; signs and symptom types and severity; and workplace and PTs’ characteristics.

### Self-care/Education

In relation to Self-care/Education, this intervention achieved mixed results. Although in the quantitative data it is not the most chosen intervention, in the qualitative data PTs considered it as the most important. One reason for this discrepancy is that PTs considered it as a mandatory intervention and should be present in all patients “since day 1”. In fact, although not often prescribed, PTs naturally performed it. As so, many of the PTs could not choose it in our e-survey, as they almost see it as a moral duty and not so much as an intervention. Nevertheless, this intervention was integrated in the 4^th^ most chosen interventions combination, being easily associated with other interventions (21 interactions in a 28 interventions sample) and used in all signs and symptoms approached. To proper perform it, it is important to adapt the information to the patients’ health literacy and provide different information supports (oral and written).^42,89^ If the PT do not adapt the information to the patient’s health literacy or provide it in just one way, the information transmitted could be lost or misunderstood. To ensure that the patient truly understand the information given, a simple test could be performed, the so-called “Kieran O’Sullivan test”. This test suggests that the PT should ask the patients to describe how they will explain the information given to their family (or significant other) when they come back to home. Evidence highly recommends its use in these patients (A)^39,41,42,45–47,50,52,53,55^ since practitioners should continually provide their patients with necessary information about: OA disease progression; knee anatomy; pathophysiology; joint protection; home exercises and self-care techniques; and overall lifestyle changes. The objective is to promote hope, optimism, and a positive expectation of the benefits of the intervention plan.^39,41,42,45,46,53,64,89^ Furthermore, during PT-patient communication, PTs should avoid using “wear and tear”, “it’s your age”, “nothing can be done for you”, or “give up” expressions, as they could result in negative feelings in the patients regarding the intervention plan and the OA progression.^90^

### Other

As shown, other interventions were applied due to personal, patient, and work-related factors. Similar factors were found in other countries and health care professions, showing that they could condition the interventions choice.^81,91,92^

In the personal factors, PTs’ age and experience may have an important role in the intervention plan design. In our study, Exercise modalities were more chosen in young and less experienced PTs. This may be explained by evidence access and given importance.^93^ Younger PTs could be more technologically advanced and could access evidence quicker compared to their older peers. Furthermore, in their intervention plan rationale, they cannot balance evidence and clinical experience equally, they have experience deficits. In other hand, older and more experienced Portuguese PTs may have less ability to reach evidence and tend to follow their clinical experience even more.^67^ In fact, it is expected that only half of the PTs use databases to aid in clinical decision-making.^93^ Additionally, personal doubts about evidence and treatment effectiveness may also exist.^91^ Explanation for this may include^93,94^: poor quality evidence; contradictory clinical practice guidelines (CPGs) recommendations; poor quality in the information transmission; PTs inability to understand statistical data; lack of skills in searching and critically appraise evidence; lack of data generalisation for the patient; and not enough explored OA factors, such as economic aspects of recommendations or the patients’ co-morbidities influence. Facilitators may include^94–95^: regular clinical cases and evidence peers discussion; higher quality studies; CPGs concordance; better information reaching with an user-friendly format; CPGs should become patient-focused rather than disease-driven. Academic degree, belonging to a practice-oriented organisation, and participate in continuing educational courses may also influence the Portuguese PT practice, however further studies are needed to understand their true importance.

Patient was a central piece on the decision-making process puzzle. Almost all PTs reported that the interventions choice was from the patients’ signs and symptoms, co-morbidities, and other clinical findings (such as pain, ROM limitations, muscular weakness, and activities of daily living restrictions). There were similar to evidence-reported most important factors.^96–98^ As the interventions are applied in the patients, the PTs also though that their preferences have an important role. Nevertheless, in a deeper analysis, the PTs used it in their clinical-making intervention plan more as a way to decide between two equal effective interventions, or as “bargaining chip” to introduce more evidence-based interventions. Therefore, a better PT-patient communication and interaction is mandatory, as well as more importance needs to be given to their preferences, providing them with a more active participation in the intervention plan decision.^79,99^ Patients are often septic and pessimist about interventions and OA progression.^63,99^ So, other factors may also be important to increase the knee OA patients’ optimism, satisfaction and security, such as^79,99^: good PT accessibility, deviation, convention, prioritising therapeutic over financial consideration; PT competence; feeling that their opinions and preferences are taken into account; and their intervention plan is individualised.

The structure of the system in which PTs worked influenced their knee OA treatment approach. In this factor, two main issues raised; money and time. As in Portugal the salaries are low and the PTs services are considered as cheap, sometimes the PTs have to give in to the patients demands (even if the PT do not agree with the intervention efficacy) as they could lose a client and consequently money (since most of the small health care units are client-financially dependent). In other hand, there are bigger health care units that are stated-financially dependent, so many times the PTs have to do what is medically prescribed and stated funded. In fact, 88–90% of the Portuguese patients with knee OA reach physical therapy after general practitioners consultation and prescription.^66,67^ Lack of money could also result in a lack of resources (such as, technological clinical equipment or computers), influencing the interventions choice.^93^ Similar to what was found in other studies,^93^ time was one of the largest work-related barriers. In our study, the PTs needed time to evaluate, review and treat patients, and for extra work activities such as evidence or skills improvements. Comparable concepts were found in UK PTs.^54^

Also, the workplace environment itself could be a barrier.^93^ One of the most important barrier to the Portuguese PTs is that in the workplace it is not given enough importance if they do (or not) an EBP.^67^ Other barriers found in the literature include^93^: lack of support from the employer; and colleagues not favourable to EBP.

### Limitations

One limitation of this study was the number of valid questionnaires. The sample size goal of 373 was not reached. Therefore, the results could not truly represent the Portuguese PTs practice. Another limitation was found in the qualitative data, where the instruments used in the patients’ evaluation and follow-up were not fully explored and understood. Finally, it would also be interesting to have conducted the study with different clinical vignettes to understand how patients’ characteristics, the level of pain, joint range of motion, functionality, physical activity, or other clinical findings influence the choice of intervention.

## CONCLUSION

In conclusion, in the context of Portuguese PTs, the most important interventions are Exercise (specially, Resistance Training), Manual Therapy, Nutrition/Weight Loss and Self-care/Education. PTs individual characteristics (age, experience, and clinical reasoning), patient’s characteristics (clinical findings and preferences), and work-related factors (facility type, work environment, and available resources) are the main actors responsible for the use (or not) of an intervention.

## Figures and Tables

**Suppl. Table 1.** Checklist for Reporting Results of Internet E-Surveys (CHERRIES).

***Checklist Item***	***Explanation***	***Page Number***
Describe survey design	Describe target population, sample frame. Is the sample a convenience sample? (In “open” surveys this is most likely.)	6
IRB approval	Mention whether the study has been approved by an IRB.	5
Informed consent	Describe the informed consent process. Where were the participants told the length of time of the survey, which data were stored and where and for how long, who the investigator was, and the purpose of the study?	6
Data protection	If any personal information was collected or stored, describe what mechanisms were used to protect unauthorized access.	-
Development and testing	State how the survey was developed, including whether the usability and technical functionality of the electronic questionnaire had been tested before fielding the questionnaire.	7
Open survey versus closed survey	An “open survey” is a survey open for each visitor of a site, while a closed survey is only open to a sample which the investigator knows (password-protected survey).	6
Contact mode	Indicate whether or not the initial contact with the potential participants was made on the Internet. (Investigators may also send out questionnaires by mail and allow for Web-based data entry.)	6
Advertising the survey	How/where was the survey announced or advertised? Some examples are offline media (newspapers), or online (mailing lists – If yes, which ones?) or banner ads (Where were these banner ads posted and what did they look like?). It is important to know the wording of the announcement as it will heavily influence who chooses to participate. Ideally the survey announcement should be published as an appendix.	6
Web/E-mail	State the type of e-survey (eg, one posted on a Web site, or one sent out through e-mail). If it is an e-mail survey, were the responses entered manually into a database, or was there an automatic method for capturing responses?	6
Context	Describe the Web site (for mailing list/newsgroup) in which the survey was posted. What is the Web site about, who is visiting it, what are visitors normally looking for? Discuss to what degree the content of the Web site could pre-select the sample or influence the results. For example, a survey about vaccination on a anti-immunization Web site will have different results from a Web survey conducted on a government Web site	6
Mandatory/voluntary	Was it a mandatory survey to be filled in by every visitor who wanted to enter the Web site, or was it a voluntary survey?	6
Incentives	Were any incentives offered (eg, monetary, prizes, or non-monetary incentives such as an offer to provide the survey results)?	6
Time/Date	In what timeframe were the data collected?	
Randomization of items or questionnaires	To prevent biases items can be randomized or alternated.	7
Adaptive questioning	Use adaptive questioning (certain items, or only conditionally displayed based on responses to other items) to reduce number and complexity of the questions.	-
Number of Items	What was the number of questionnaire items per page? The number of items is an important factor for the completion rate.	-
Number of screens (pages)	Over how many pages was the questionnaire distributed? The number of items is an important factor for the completion rate.	-
Completeness check	It is technically possible to do consistency or completeness checks before the questionnaire is submitted. Was this done, and if “yes”, how (usually JAVAScript)? An alternative is to check for completeness after the questionnaire has been submitted (and highlight mandatory items). If this has been done, it should be reported. All items should provide a non-response option such as “not applicable” or “rather not say”, and selection of one response option should be enforced.	-
Review step	State whether respondents were able to review and change their answers (eg, through a Back button or a Review step which displays a summary of the responses and asks the respondents if they are correct).	7
Unique site visitor	If you provide view rates or participation rates, you need to define how you determined a unique visitor. There are different techniques available, based on IP addresses or cookies or both.	8
View rate (Ratio of unique survey visitors/unique site visitors)	Requires counting unique visitors to the first page of the survey, divided by the number of unique site visitors (not page views!). It is not unusual to have view rates of less than 0.1 % if the survey is voluntary.	10
Participation rate (Ratio of unique visitors who agreed to participate/unique first survey page visitors)	Count the unique number of people who filled in the first survey page (or agreed to participate, for example by checking a checkbox), divided by visitors who visit the first page of the survey (or the informed consents page, if present). This can also be called “recruitment” rate.	10
Completion rate (Ratio of users who finished the survey/users who agreed to participate)	The number of people submitting the last questionnaire page, divided by the number of people who agreed to participate (or submitted the first survey page ). This is only relevant if there is a separate “informed consent” page or if the survey goes over several pages. This is a measure for attrition. Note that “completion” can involve leaving questionnaire items blank. This is not a measure for how completely questionnaires were filled in. (If you need a measure for this, use the word “completeness rate”.)	10
Cookies used	Indicate whether cookies were used to assign a unique user identifier to each client computer. If so, mention the page on which the cookie was set and read, and how long the cookie was valid. Were duplicate entries avoided by preventing users access to the survey twice; or were duplicate database entries having the same user ID eliminated before analysis? In the latter case, which entries were kept for analysis (eg, the first entry or the most recent)?	-
IP check	Indicate whether the IP address of the client computer was used to identify potential duplicate entries from the same user. If so, mention the period of time for which no two entries from the same IP address were allowed (eg, 24 hours). Were duplicate entries avoided by preventing users with the same IP address access to the survey twice; or were duplicate database entries having the same IP address within a given period of time eliminated before analysis? If the latter, which entries were kept for analysis (eg, the first entry or the most recent)?	8
Log file analysis	Indicate whether other techniques to analyze the log file for identification of multiple entries were used. If so, please describe.	-
Registration	In “closed” (non-open) surveys, users need to login first and it is easier to prevent duplicate entries from the same user. Describe how this was done. For example, was the survey never displayed a second time oncethe user had filled it in, or was the username stored together with the survey results and later eliminated? If the latter, which entries were kept for analysis (eg, the first entry or the most recent)?	-
Handling of incomplete questionnaires	Were only completed questionnaires analyzed? Were questionnaires which terminated early (where, for example, users did not go through all questionnaire pages) also analyzed?	10
Questionnaires submitted with an atypical timestamp	Some investigators may measure the time people needed to fill in a questionnaire and exclude questionnaires that were submitted too soon. Specify the timeframe that was used as a cut-off point, and describe how this point was determined.	-
Statistical correction	Indicate whether any methods such as weighting of items or propensity scores have been used to adjust for the non-representative sample; if so, please describe the methods.	-

This checklist has been modified from Eysenbach G. Improving the quality of Web surveys: the Checklist for Reporting Results of Internet E-Surveys (CHERRIES). J Med Internet Res. 2004 Sep 29;6(3):e34 [erratum in J Med Internet Res. 2012; 14(1): e8.]. Article available at https://www.jmir.org/2004/3/e34/ ; erratum available https://www.jmir.org/2012/1/e8/. Copyright ©Gunther Eysenbach. Originally published in the Journal of Medical Internet Research, 29.9.2004 and 04.01.2012.

This is an open-access article distributed under the terms of the Creative Commons Attribution License (https://creativecommons.org/licenses/by/2.0/), which permits unrestricted use, distribution, and reproduction in any medium, provided the original work, first published in the Journal of Medical Internet Research, is properly cited.

**Suppl. Table 2.** COREQ (COnsolidated criteria for REporting Qualitative research) Checklist. A checklist of items that should be included in reports of qualitative research. You must report the page number in your manuscript where you consider each of the items listed in this checklist. If you have not included this information, either revise your manuscript accordingly before submitting or note N/A.

**Topic**	**Item No.**	**Guide Questions/Description**	**Reported on Page No.**
**Domain 1: Research team and reflexivity**			
*Personal characteristics*			
Interviewer/facilitator	1	Which author/s conducted the interview or focus group?	8
Credentials	2	What were the researcher’s credentials? E.g. PhD, MD	8
Occupation	3	What was their occupation at the time of the study?	8
Gender	4	Was the researcher male or female?	8
Experience and training	5	What experience or training did the researcher have?	8
*Relationship with participants*			
Relationship established	6	Was a relationship established prior to study commencement?	8
Participant knowledge of the interviewer	7	What did the participants know about the researcher? e.g. personal goals, reasons for doing the research	8
Interviewer characteristics	8	What characteristics were reported about the inter viewer/facilitator? e.g. Bias, assumptions, reasons and interests in the research topic	8
**Domain 2: Study design**			
*Theoretical framework*			
Methodological orientation and Theory	9	What methodological orientation was stated to underpin the study? e.g. grounded theory, discourse analysis, ethnography, phenomenology, content analysis	9
*Participant selection*			
Sampling	10	How were participants selected? e.g. purposive, convenience, consecutive, snowball	8
Method of approach	11	How were participants approached? e.g. face-to-face, telephone, mail, email	8
Sample size	12	How many participants were in the study?	12
Non-participation	13	How many people refused to participate or dropped out? Reasons?	12
*Setting*			
Setting of data collection	14	Where was the data collected? e.g. home, clinic, workplace	8
Presence of non-participants	15	Was anyone else present besides the participants and researchers?	8
Description of sample	16	What are the important characteristics of the sample? e.g. demographic data, date	12
*Data collection*			
Interview guide	17	Were questions, prompts, guides provided by the authors? Was it pilot tested?	8
Repeat interviews	18	Were repeat inter views carried out? If yes, how many?	-
Audio/visual recording	19	Did the research use audio or visual recording to collect the data?	8
Field notes	20	Were field notes made during and/or after the inter view or focus group?	-
Duration	21	What was the duration of the inter views or focus group?	12
Data saturation	22	Was data saturation discussed?	-
Transcripts returned	23	Were transcripts returned to participants for comment and/or correction?	-

**Topic**	**Item No.**	**Guide Questions/Description**	**Reported on Page No.**
**Domain 3: analysis and findings**			
*Data analysis*			
Number of data coders	24	How many data coders coded the data?	12
Description of the coding tree	25	Did authors provide a description of the coding tree?	12
Derivation of themes	26	Were themes identified in advance or derived from the data?	9
Software	27	What software, if applicable, was used to manage the data?	9
Participant checking	28	Did participants provide feedback on the findings?	-
*Reporting*			
Quotations presented	29	Were participant quotations presented to illustrate the themes/findings? Was each quotation identified? e.g. participant number	12
Data and findings consistent	30	Was there consistency between the data presented and the findings?	12
Clarity of major themes	31	Were major themes clearly presented in the findings?	12
Clarity of minor themes	32	Is there a description of diverse cases or discussion of minor themes?	12

Developed from: Tong A, Sainsbury P, Craig J. Consolidated criteria for reporting qualitative research (COREQ): a 32-item checklist for interviews and focus groups. *International Journal for Quality in Health Care*. 2007. Volume 19, Number 6: pp. 349 – 357

**Once you have completed this checklist, please save a copy and upload it as part of your submission. DO NOT include this checklist as part of the main manuscript document. It must be uploaded as a separate file.**

**Suppl. Table 3.** PTs’ Intervention choices.

**Interventions**	**Response options (n; %)**						

	*1º*	*2º*	*3º*	*4º*	*5º*	Total
**Balneotherapy/Spa**	0 (0.0%)	0 (0.0%)	0 (0.0%)	0 (0.0%)	0 (0.0%)	0 (0.0%)
**Bandages**						
Elastic Tape	0 (0.0%)	1 (7.7%)	2 (15.4%)	3 (23.1%)	7 (53.8%)	13 (2.2%)
Non-elastic Tape	0 (0.0%)	0 (0.0%)	0 (0.0%)	1 (25.0%)	3 (75.0%)	4 (0.7%)
**Braces**	0 (0.0%)	0 (0.0%)	0 (0.0%)	0 (0.0%)	1 (100.0%)	1 (0.2%)
**Complementary Therapies**						
Acupuncture	1 (25.0%)	1 (25.0%)	2 (50.0%)	0 (0.0%)	0 (0.0%)	4 (0.7%)
Cupping Therapy	0 (0.0%)	0 (0.0%)	0 (0.0%)	0 (0.0%)	0 (0.0%)	0 (0.0%)
Electroacupuncture	1 (25.0%)	3 (75.0%)	0 (0.0%)	0 (0.0%)	0 (0.0%)	4 (0.7%)
Leech Therapy	0 (0.0%)	0 (0.0%)	0 (0.0%)	0 (0.0%)	0 (0.0%)	0 (0.0%)
Moxibustion	1 (33.3%)	1 (33.3%)	0 (0.0%)	0 (0.0%)	1 (33.3%)	3 (0.5%)
Tai Ji	0 (0.0%)	0 (0.0%)	0 (0.0%)	0 (0.0%)	1 (100.0%)	1 (0.2%)
Yoga	0 (0.0%)	0 (0.0%)	0 (0.0%)	1 (50.0%)	1 (50.0%)	2 (0.3%)
**Electrical Stimulation Therapy**						
Interferential Current	3 (25.0%)	1 (8.3%)	6 (50.0%)	1 (8.3%)	1 (8.3%)	12 (2.0%)
NMES	1 (8.3%)	2 (16.7%)	4 (33.3%)	3 (25.0%)	2 (16.7%)	12 (2.0%)
TENS	2 (22.2%)	1 (11.1%)	3 (33.3%)	0 (0.0%)	3 (33.3%)	9 (1.5%)
**Exercise Therapy**						
Aerobic Exercise	2 (9.1%)	5 (22.7%)	8 (36.4%)	4 (18.2%)	3 (13.6%)	22 (3.7%)
Aquatic Exercise	7 (15.2%)	8 (17.4%)	7 (15.2%)	7 (15.2%)	17 (37.0%)	46 (7.7%)
Balance Exercise	3 (7.7%)	4 (10.3%)	12 (30.8%)	10 (25.6%)	10 (25.6%)	39 (6.5%)
Resistance Exercise	12 (13.8%)	25 (28.7%)	23 (26.4%)	18 (20.7%)	9 (10.3%)	87 (14.5%)
Stretching	1 (2.1%)	9 (19.1%)	8 (17.0%)	17 (36.2%)	12 (25.5%)	47 (7.8%)
Vibration	0 (0.0%)	0 (0.0%)	1 (100.0%)	0 (0.0%)	0 (0.0%)	1 (0.2%)
**Extracorporeal Shockwave Therapy**	0 (0.0%)	0 (0.0%)	1 (50.0%)	1 (50.0%)	0 (0.0%)	2 (0.3%)
**Insoles**	0 (0.0%)	1 (25.0%)	1 (25.0%)	2 (50.0%)	0 (0.0%)	4 (0.7%)
**Laser Therapy**						
High Level Laser Therapy	1 (20.0%)	1 (20.0%)	0 (0.0%)	0 (0.0%)	3 (60.0%)	5 (0.8%)
Low Level Laser Therapy	0 (0.0%)	1 (25.0%)	0 (0.0%)	2 (50.0%)	1 (25.0%)	4 (0.7%)
**Magnetic Field Therapy**	1 (20.0%)	1 (20.0%)	2 (40.0%)	1 (20.0%)	0 (0.0%)	5 (0.8%)
**Manual Therapy**	37 (43.0%)	21 (24.4%)	15 (17.4%)	7 (8.1%)	6 (7.0%)	86 (14.3%)
**Nutrition Therapy/Weight Loss**	16 (19.5%)	19 (23.2%)	11 (13.4%)	19 (23.2%)	17 (20.7%)	82 (13.7%)
**Self-care/Education**	25 (42.4%)	5 (8.5%)	6 (10.2%)	9 (15.3%)	14 (23.7%)	59 (9.8%)
**Thermal Agents**	2 (14.3%)	3 (21.4%)	2 (14.3%)	4 (28.6%)	3 (21.4%)	14 (2.3%)
**Ultrasonic Therapy**	1 (5.3%)	5 (26.3%)	4 (21.1%)	7 (36.8%)	2 (10.5%)	19 (3.2%)
**Walking Aids**	3 (23.1%)	2 (15.4%)	2 (15.4%)	3 (23.1%)	3 (23.1%)	13 (2.2%)

**Suppl. Table 4.**
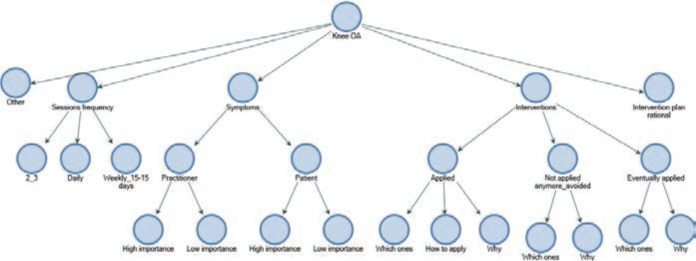
Classification tree.

**Suppl. Table 5.** PTs’ characteristics in the qualitative study.

**Characteristics**	**Physical Therapists**										
	PT 1	PT 2	PT 3	PT 4	PT 5	PT 6	PT 7	PT 8	PT 9	PT 10
Sex	Male	Female	Female	Male	Male	Female	Male	Female	Female	Female
Age	20–29	20–29	20–29	40–49	< 50	30–39	30–39	30–39	20–29	30–39
Years of License	> 5	> 5	> 5	< 15	< 15	11–15	5–10	5–10	5–10	11–15
Academic Degree	Bac.	Bac.	Bac.	Doc.	Mas.	Bac.	Mas.	Mas.	Bac.	Bac.
Working Hours	31–40	20–30	20–30	< 40	< 40	31–40	< 40	31–40	< 40	31–40
Patients Day	6–10	6–10	11–15	6–10	1–5	11–15	6–10	11–15	11–15	6–10
PTs in the Facility	1–5	1–5	1–5	< 15	0	1–5	0	6–10	6–10	0
% Time in:										
*Patient Care*	80–100%	55–75%	55–75%	30–50%	5–25%	80–100%	55–75%	30–50%	80–100%	80–100%
*Researcher*	5–25%	5–25%	30–50%	5–25%	5–25%	0%	5–25%	5–25%	0%	5–25%
*Teacher*	0%	0%	0%	55–75%	80–100%	0%	30–50%	5–25%	0%	0%
Majority of Patients	Adult	Geriatric	Geriatric	Geriatric	Adult	Geriatric	Adult	Adult	Adult	Adult
Work Sector	Private	Private	Public	Academic	Academic	Private	Private	Public	Private	Private
Work Mode	Others Account	Own Account	Own Account	Others Account	Others Account	Others Account	Own Account	Others Account	Others Account	Own Account

Bac.: Baccalaureate; Mas.: Master; Doc.: Doctorate.

All included PTs pursue a higher academic degree, participate in continuing education courses, and belong to a practice-orientated organisation.

**Suppl. Table 6.** Quotations. Interventions applied

The stated applied interventions to manage knee OA patients were: Self care/Education; Electrical Therapy; Exercise; Kinesio Tape (KT); Manual Therapy; Thermal Agents; US; and Vertical Bed. The PTs’ choose to use these interventions because: (1) after the patient assessment, they are the interventions that best respond to the patients’ signs and symptoms, as well as the treatment objectives created; (2) are in accordance to an EBP; and, (3) give priority to more active interventions (Quotations 1, 2 and 3).
*Quotation 1: “... I try to apply interventions that give movement to the knee, trying in some way to respect the symptoms and using strategies to reduce them ... (...) I give priority to evidence based interventions.” [FT_1]*
*Quotation 2:” Always according to the initial assessment made and the patient’s signs and symptoms, obviously. (…) Usually, we do the subjective examination – the subjective examination is extremely important – i. e., the patient tells us exactly his problems and then we will try to transform this into compatible signs. In the case of an elderly OA person, the structure tests don’t seem to be the most important, for me. Here it’s easy to see if there’s a change in alignment, other changes in dimension, etc., that may appear during the mobility of the structure. But I focus a lot and worry a lot about the patient’s activities and participation. And that’s when I start to do my objective tests and start by observing the gait, watching walking the stairs up and down, asking how he/she does his day to day chores, dressing, undressing, going to bathroom, all activities, etc., in which the knee may disturb. Within activities, that’s where I worry. That’s when I’m going to be very concerned.” [FT_4]*
*Quotation 3: “… it’s much more a question of I clinically realizing that maybe the results offered are not that good, and a question of evidence. Regarding that second possibility [patients], despite at this moment it doesn’t happen so much (although it does happen from time to time), but the patients still come with the idea of conventional physical therapy and be a little reticent when we show that what we do here is a little different … the work is different. They are waiting for a purely passive treatment and, suddenly, I show them by ‘a + b’ that there are other more active strategies with better long term results and that can help them more. And patients often preferred to arrive, lie down on the table and someone will treat them. And a lot of the work I do is, initially, explain and show why is important to do something else … to take another approach. In other words, it’s not the patients’ preference that led me to change this, but the question of evidence and the question of considering that in the short, medium and long term the results are better with other techniques.” [FT_7]*
In relation to Self-care/Education, it was performed with the aim of: (1) informing the patient about its condition, OA related issues and intervention plan; (2) ending OA related myths; (3) explaining how to manage their problems alone, including giving some home interventions that could help improving their condition; (4) promoting an healthy life style, such as weight loss; and, (5) during all treatments sessions, explaining how to perform the intervention properly and why they are doing it (Quotations 4, 5, 6, 7 and 8). As a note, a PT added that it is important to adapt the information to the patient’s health literacy/academic qualifications, for him/her to receive and better understand the transmitted information (Quotation 9).
*Quotation 4: “Even, sometimes, prove to the person that he/she can move and should move the knee without pain… that the fact of having knee OA isn’t: ‘Oh! This is a chronic problem and now I will have knee pain for life.’ It’s necessary to deconstruct this myth and prove that it’s possible to move the knee and walk without pain. (…) ... education is present from the beginning to the end. Sometimes isolated, but I try whenever it’s possible to associate education with movement. While the person is moving, or while resting in a certain exercise: ‘Oh, you see, we did this, we did that’.” [FT_1]*
*Quotation 5: “... in many cases in people with pain and kinesiophobia it’s important to educate the person ... however, often, it’s not prescribed …” [FT_2]*
*Quotation 6: “So I have to rely a lot on the patient’s educational model. I have to teach how to do exercises at home. It’s not enough to do the intervention at the clinic. (…) Therefore, my practice goes a lot with the patient’s education to avoid risk factors, overweight, immobility and exercise instruction.” [FT_5]*
*Quotation 7: “But, of course, in these people ... we try to privilege the increase of physical activity in general, because they are usually coming with the idea that their knees are like ‘tires’, i. e., the more they walk, the more they wear out. Deconstructing these ideas, trying to increase the level of physical activity globally, and gradually exposing them to more specific lower limbs exercises ... (…) … simple things, like some tips in sleep hygiene, some education in relation to fear of movement, and then the question of increasing activity levels and specific exercises” [FT_7]*
*Quotation 8: “Especially in the beginning of education, people often come with the belief that it’s ‘massage, heat, and electrotherapy’… and, many times, people come with bad beliefs that ‘they can’t move because they are going to get worse’ and, throughout the treatments, what I tried to do is demystify it. If the person actually had these kinds of beliefs. (…) And for knee OA, care should all be done in primary care, with exercise, with education to people, with explanation to people of what they should do to prevent the progression of the disease or, at least, to maintain functionality. (...) Yes, no doubt. People will receive treatment for knee OA completely misguided. People’s beliefs are always ointments and surgery. It never drifts that much. Because: ‘if it hurts we have to be still’.” [FT_8]*
*Quotation 9: “Q: Do you educate the patient for his own pathology?*
*Yes. And according to the patient’ health literacy/academic qualifications in general (which often end up being a limitation). I always explain and believe that patients always want to know what they have, what the prognosis is and what is expected to happen there. Therefore, sometimes going into very scientific details about the etiology of the problem and everything, it’s possible with some metaphors to briefly explain this, what people most want to know: ‘Will I stay like this forever?’; ‘How can I improve?’; ‘What can I do?’; ‘What can’t I do?’. And then yes, I explain … I always try to find time, either at the beginning or at the end of the session to do it. Yes, education is important.” [FT_1]*
In the Electrical Therapy group, TENS, Iontophoresis, IFC and NMES were used. Except for NMES, all Electrical Therapy group interventions were applied to reduce pain (Quotations 10 and 11). Between the different analgesic techniques, their choices depended on: (1) what the interventions that health insurers companies contributed financially were; and, (2) which medical devices were present at the health care units (Quotations 12 and 13). Despite this, two PTs were against their use, since their benefits were limited and there is some evidence non-recommendation (Quotation 14). In other hand, NMES was the least stated intervention in this group and it was mainly used as a complementation of active exercises, or as an initiation stage for more debilitated patients (not able to perform active exercises) to gain some strength (Quotations 15 and 16).
*Quotation 10: “Then some electrical therapy analgesic techniques, such as iontophoresis, US and TENS. That’s what we usually do.” [FT_4]*
*Quotation 11: “... then, some electrophysical therapeutic means for pain control, namely IFC or TENS. I don’t go much further than that and only with the analgesic objective.” [FT_5]*
*Quotation 12: “... I have to respect the prescription, because the act is paid by the health insurance company ... being TENS, massage, among others.” [FT_2]*
*Quotation 13: “Yes, thermotherapy, radiofrequency… I’m thinking of the devices that we have ...*
*Q: Laser, is also very ...*
*No … at the moment, we don’t have a laser at the clinic. The only devices we have are TENS, radiofrequency and shock waves.” [FT_9]*
*Quotation 14: “... there’s a very small percentage of patients who actually feel relief ... and that takes a lot of session time, for the gains that they have. And, at this moment, I don’t use it, because there was a recommendation for not using it at all or against TENS use.” [FT_8]*
*Quotation 15: “I tend to use it less to reduce pain. I use it as a form of strengthening, but from the moment he/she can do it without help, he/she does it actively” [FT_7]*
*Quotation 16: “In some cases, if we see that there was already a decrease, even in terms of musculature, we tried to use NMES for strengthening together with exercises, at the same time. This would be more for the increase of the musculature.” [FT_9]*
Exercises were the most frequent intervention used for managing knee OA patients. The interventions stated in this group were: Aerobic exercises; Active mobilizations/Rage of Motion (ROM) gain/Stretching exercises; Resistance exercises; and Functional training/exercises. From all, Resistance exercises were the most used, mainly because of OA progression characteristics (namely, strength loss). Within these exercises, the PTs preferred to start performing isometric exercises, only progressing to other contractions types and exercises after the patients reveal a good strength standard base (Quotation 17). Regarding the Aerobic exercises, they were performed to promote knee movement, reduce impacts, as well as maintain the physical condition, being then a form of preparation for more intense exercises (Quotation 18). With similar objectives were the Active mobilizations/ROM gain/Stretching exercises. The PTs used these exercises not only to increase (or at least maintain) a “normal” knee ROM, but also to decrease the muscular and articular tensions (Quotations 19, 20 and 21). Lastly, they also reported the use of Functional training/exercises. These exercises were applied to train the activities of daily life (such as, stand and sit, stability training and gait training), equipping/educating the patient with a range of strategies to reduce some daily constraints (Quotation 22). The intervention choices in the Exercise group depended, not only on the patients’ condition and the OA degree (such as, obesity, ROM limitation and knee inflammation), but most importantly pain. PTs evaluated their patients’ pain level and in which exercises the pain intensified more. The aim was always to do pain free exercises, giving to the patients a good movement experience and sensation (Quotation 23).
*Quotation 17: “We usually do strength exercises, yes. It will depend on the patients’ condition and complaints. We can do isometric exercises or we can then progress to slightly different ones. But yes, they are usually strength exercises.” [FT_3]*
*Quotation 18: “So, squats, in an acute situation, I never done it. Because it was loaded. I try to do more muscle strengthening exercises in an open kinetic chain at an early stage. Or without load, to try to minimize the impacts and then yes, progress to more loading exercises.*
*Q: Regarding unloaded exercises is it, for example, stationary bike? Or do you never used this strategy?*
*I also do the elliptical sometimes. Although it depends. In the nursing home, I do a lot of elliptical exercises because they don’t have to do much knee flexion. And pedalboard … I also use a lot of pedalboard.” [FT_10]*
*Quotation 19: “I usually use unloading active exercise training to increase amplitudes (always active techniques).” [FT_4]*
*Quotation 20: “... I also do a lot of muscle chains stretching to relieve tension (myotensive). I stretch the entire muscle chain and this will lessen joint tension. (...) This is more based on experience. But the fact is that when I start working on the flexibility in a more global perspective, with muscle chains, every OA patients improves. Sometimes, I don’t start it in the first sessions. I begin first with more active mobilizations. But after 2 or 3 sessions, when I start to introduce a more serious myotensive work, with more flexibility, it’s when I see more improvements. It’s interesting!” [FT_5]*
*Quotation 21: “Besides education, I use mobilization with stretches, in an attempt to maintain joint amplitudes.” [FT_8]*
*Quotation 22: “… and then functional training (from the basics to sitting and getting up, standing and sitting, gait training) ...” [FT_1]*
*Quotation 23: “... of course it depends on the stage, the overall health condition and the pain degree. (...) ... promote low load movement initially and as pain free as possible. Create a good movement experience for the patient with movements that are important for him/her (activities of daily living, such as walking, standing, sitting, squatting, etc.).” [FT_1]*
The use of KT was only indicated by 2 PTs. In both cases, although it was used by them, none considered it to be a core intervention. The intervention was considered as complementary. Additionally, in both situations, they were somewhat reticent and septic about its clinical efficacy. The main reason for this mistrust was due to the results found in evidence, which often discredited its use and deconstructed some efficacy related-myths. Nevertheless, its use was mainly for pain relief and knee biomechanical corrections (Quotations 24 and 25).
*Quotation 24: “There are always new and trendy interventions … like KT. There was a time when a lot of people with OA were walking with KT. Nowadays, within the various limitations that Kinesio has, we often get relief from symptoms immediately by repositioning the patella, when the problem is in the patellofemoral compartment. And so, sometimes it can be a good strategy for immediate symptom relief. When a person gets there with pain he/she always goes a little better after it. Now, a patient will not always walk with that. It’s necessary to do another type of work to help to give more quality of live....” [FT_1]*
*Quotation 25: “Then there are things that sometimes surprise us … like KT. I forgot to mention that in these situations [knee OA patients] I apply KT. (...)*
*Q: Are you saying it’s a placebo?*
*Yeah…but today I don’t say that. I don’t say that anymore, because there’s already some evidence. I think that some things that are said about KT are a lie, and in the studies that I developed with undergraduate and master students in the institution where I am, we have been proving that some things that Mr. Kenso Kase said don’t correspond to the truth. But, in terms of clinical effectiveness, in some cases it’s very interesting … I don’t know if it’s a placebo, but in fact it’s very interesting.” [FT_5]*
Likewise Exercise, Manual Therapy was one of the most intervention groups mentioned and used by the PTs. Within this group, the most common interventions applied were: Massage; Passive mobilizations; and, Soft tissues mobilization/manipulation techniques (Quotations 26 and 27). Additionally, Maitland and Mulligan techniques were also referred in this group, however just one PT confirmed their use (Quotation 28). The overall objectives of these interventions were soft tissue relaxation (harmonize muscular tensions, stabilize muscular tonus and release soft tissues), drainage, pain decrease and ROM improvement (Quotation 29). Nevertheless, one PT did not recommend the use of Massage on these patients, because it will not offer any of the overall intended benefits, which are providing mobility and functionality (Quotation 30). Additionally, another PT do not recommend the use of Passive mobilization. He/She preferred to spend most of the time doing more active interventions (Quotation 31).
*Quotation 26: “… manual therapy like massage or physiological and accessory mobilizations …” [FT_3]*
*Quotation 27: Objectively, when I use much more specific techniques, like tonus reduction, tissue release … they are very localized techniques for that moment … for the mobilization of soft tissues ...” [FT_4]*
*Quotation 28: “… I’m not an osteopath, but I’m a manual therapist. I use a lot of manual therapy … (…) But – if you want me to be very concrete – I use Maitland techniques for accessory movements, for gaining amplitude and pain relief, and I use Mulligan techniques with mobilization with movement…” [FT_5]*
*Quotation 29: “Q: What are the goals of manual therapy?*
*It would be more at the level of relaxation. Because there are always some muscle tensions, more on one side than the other. Harmonize muscle tensions and to help in some inflammation.*
*Q: So, drainage of some edema that could be present. And pain, no?*
*Yes. that too.” [FT_9]*
*Quotation 30: “I think that for the treatment purposes of today, massage doesn’t meet the real treatment goals. If we want more mobility and functionality of the patient, he/she has to be active and participative. So it’s not massage that will solve it. For that, he/she needs action, he/she needs to participate. (...) I think that massage no longer responds to the needs that we have.” [FT_4]*
*Quotation 31: “... passive mobilizations (although sometimes I still do it, I try to do it as physiological and active possible).” [FT_1]*
Although the PTs use Thermal Agents, this intervention group was far from gathering consensus. In this group, the two interventions stated were Heat and Ice. All PTs considered Thermal Agents to be a complementary intervention. In fact, most of them just use it because the patient asks for it (Quotation 32). Their use depended on the patient’s condition, nonetheless the aim was to decrease pain (Heat and Ice), promote muscular relaxation (Heat) and reduce the inflammatory process (Ice) (Quotations 33, 34 and 35). Despite these advantages, some PTs did not recommend their use. For the Heat interventions, they did not use them because it could increase the knee inflammation process (Quotation 36). Regarding the Ice interventions, the PTs said that it could increase some of the patients’ signs and symptoms (Quotation 37).
*Quotation 32: “For example heat. Although at this point I end up doing it, I only do it for the patient’s preferences. The patient asks it and sometimes it’s necessary to give him/her certain things in order to have greater confidence and take him/her on other paths ...” [FT_1]*
*Quotation 33: “… we often use some kind of heat, which helps with stiffness, helps to reduce muscle tension, and facilitates the work that we do afterwards…” [FT_3]*
*Quotation 34: “I use ice for exaggerated inflammatory processes.” [FT_6]*
*Quotation 35: “... sometimes people like heat or ice and, at the end of the sessions, I chose to apply what the patient said most relieved him/her.” [FT_8] Quotation 36: “I once read in a study that the knee was already a joint, very prone to fluids. So if we put heat on it we would increase the amount of fluids and we could be harming instead of benefiting.” [FT_6]*
*Quotation 37: “Yes. It’s interesting, because there’s a group of OA patients that gets worse with the cold. Makes a reaction to the cold. Despite being in an inflammatory process, interesting, ‘isn’t it?’” [FT_5]*
Likewise, US were used by the PTs, however there was some reluctance in its use. In fact, some stated that they only use it because they have to follow the medical prescription (Quotation 38). The main objective was pain reduction (Quotation 39).
*Quotation 38: “Yes, I use others. That, I’m being very honest, I only do it because it’s part of it. In other words, I have a medical prescription that although isn’t fixed, we have to respect it. In other words, we have to do everything that is prescribed. (...) ... but often laser, US and electrical therapy are also prescribed …” [FT_3]*
*Quotation 39: “... or simple TENS, US and massage, basically what I do for pain.” [FT_10]*
Vertical Bed use was only stated by one PT. The intention of its use is for lower limb stretching, load stimulus and imposing knee extension. This could be important for patients who cannot perform exercises or have an excessive knee flexion (Quotation 40).
*Quotation 40: “We also use verticalization a lot. (…) … despite being able to load, they do it with an exaggerated knee flexion (if not on an inclined plane). We also often use verticalization to stretch, to give the load stimulus.” [FT_6]*
**Interventions Eventually Applied**
Still, some interventions were mentioned as interventions that could be applied in very specific situations or could eventually be included in future intervention plans. Those interventions were: Acupuncture; Aquatic exercises; McConnell Tape; Tai Ji; and Yoga (Quotations 41, 42 and 43).
*Quotation 41: “... even the McConnell Tape that I already used in knee OA ...” [FT_1]*
*Quotation 42: “... it always depends on the evaluation, but I don’t use the pool because we don’t have it…I don’t have it available where I work. However, I have already worked with some patients who used the pool in another place, articulating what they did there with another PT or exercise professional who usually follows them.” [FT_7]*
*Quotation 43: “In relation to Yoga and Tai Chi, and modalities that are a little more active and that distract the patient in some way … (...) … it may make sense for patients with pain and with important psychosocial factors and central awareness. (...) … if it helps patients to disconnect a little bit of the pain itself, the condition itself. I think that this may make sense and maybe even, perhaps, recommend to the patient as an adjunct to physical therapy, to do this type of sessions. (...) … but it’s more like as adjunct therapies, in selected patients. There isn’t really strong evidence about them…but okay. Then, in relation to patients’ preferences, there are patients who felt very good with acupuncture (personally, I don’t do acupuncture, I’m a PT and at this moment acupuncture isn’t part of the physical therapy scientific profession).” [FT_8]*
The inclusion of these interventions was highly dependent on: (1) the intervention cannot harm the patient; (2) previously, the patient needs to have a good intervention experience; (3) interventions need to have some evidence support; (4) in the absence of results from more evidence-based interventions, PTs could try these interventions; (5) has to be recommended by colleagues; (6) PTs have to do prior intervention training; and, (7) the intervention effects and objectives rationale must be plausible and appropriate (Quotations 44, 45, 46 and 47).
*Quotation 44: “If in fact the patient had a good experience with that and if it wouldn’t harm I could try it, in the failure of the most recommended treatment. (...) ... if colleagues said ‘oh, this intervention relief in the symptoms ... it worked for me’, if the evidence supports and the practice that I used don’t produce the expected results, it could be a way of trying to produce results…so yes, I would use it. Either way, I would consider other types of strategies, like referral to another professional or reassessment to see if something hadn’t been done so well or other red flags that could better indicate another condition. So, in the failure of the first line interventions, after a better patient exploration and after trying other things that may helped, I would introduce a technique that, despite not being properly validated, had good recommendations from colleagues.” [FT_1]*
*Quotation 45: “Of course, I would be able to use them if there was scientific evidence that shows me that they are being discussed or used. And that they could be suitable for my patient. I. e., not all patients accept the same intervention techniques. I also try to make a selection of the interventions to use on each patient and I can try and even be able to use it, if I know some new intervention on which I can base, which I can understand the principles and that there’s some research around it. I can experiment to use it and understand the real results. I wouldn’t use any intervention that I don’t know that it’s being studied scientifically (although the evidence may be low). Because here, the problem with physical therapy, is the evidence level … the strength of the evidence. Because the evidence is there, but the evidence strength it’s not always the best.” [FT_4]*
*Quotation 46: “It must have a plausible rational. Although studies may be lacking, if there’s an important and strong physiological basis of that treatment type have and, at least, that it doesn’t have any kind of major contraindication or that may harm patients in some way, in very selected patients, I may consider its use. (…) But effectively the patient have to already tried it and felt very good at the time ... maybe I will not recommend it but I have an open mind to say that: ‘Ok, you can try it’.” [FT_8]*
*Quotation 47: “I would use it, or at least try it, if I have enough training for that. I am very reticent about these things. I don’t do something that I don’t know 100% how to do it. First, I have to study it, I would have to be informed for that.” [FT_10]*
**Interventions Not Applied**
As it can be deduced, there was no consensus on the ideal intervention plan for these patients. Some interventions chosen by one PT could be rejected by another PT and vice-versa. In addition to the aforementioned interventions (such as, Electrical Therapy, Passive Mobilization, Thermal Agents and US), the PTs also mentioned Cupping Therapy, Laser Therapy, Magnetotherapy, Manipulation, Myocrochetage, Radiofrequency Therapy, Shockwave Therapy (Quotations 48, 49, 50 and 51).
*Quotation 48: “… laser, US, electrical therapy ....” [FT_3]*
*Quotation 49: “... there are things that were close to ‘witchcraft’, namely magnetotherapy. I really stopped using it.” [FT_5]*
*Quotation 50: “... cupping therapy, manipulations, myocrochetage, electrical therapy ... (…) ... but the ones I have listed, yes, I tend to avoid them.” [FT_7]*
*Quotation 51: “... radiofrequency and shockwave therapy aren’t used in case of OA.” [FT_9]*
In addition to the already mentioned reasons in the previous section, the PTs did not use or stopped using because they: (1) are not evidence-based; (2) do not bring enough benefits to the patient; (3) are not a priority; and, (4) the PTs are septic in believing that the clinical results are a consequence of their use (Quotations 52, 53, 54 and 55).
*Quotation 52: “… they aren’t evidence-based …” [FT_1]*
*Quotation 53: “So, in my opinion, they aren’t essential, they are adjuvants, so to speak. If I could do ‘X’ treatments, I wouldn’t prioritize these.” [FT_3]*
*Quotation 54: “And you’re not going to ask me for lasers and stuff, because I don’t believe in any of that, ‘okay?’” [FT_5]*
*Quotation 55: “... I don’t use it right now. Because I consider that ‘one way or the other’ either doesn’t work at all (I didn’t see results), or I found better strategies ...” [FT_7]*
Although these therapies are not widely used by PTs, some still used them because: (1) they are placebo and do not harm the patient; (2) the patient asks for it; and (3) there is a medical prescription and an obligation to perform them (Quotations 56 and 57). Nevertheless, sometimes it is important to use some of these interventions as they could work as an “open door” to other interventions. As the PT gives in on these less evidence-based interventions desired by the patient, the patient-PT confidence rise and then he/she can negotiate with the patient with other more evidence-based interventions, integrating them into the treatment plan and making more effective sessions.
*Quotation 56: “… there are interventions that aren’t evidence-based, but many times the patient feels better, or asks for it, and often comes with this stigma. And often I end up giving in to the beginning and then go on to work for more active strategies ...” [FT_1]*
*Quotation 57: “Yes, the physiatrist prescribes it. Although I also noticed some curious things, like … the patient’s beliefs also count, and whether I agree or not, there’s also the placebo effect associated with the thing and … there are many of the elderly who believe that laser is good. If through the placebo effect helps, since I have to do it, ok! ... that’s fine for me. There are patients who specifically say they feel better, and ‘why?’…‘maybe it was because of the laser.’” [FT_3]*
**Other**
The PTs reported that, whenever possible, they used an EBP to define the knee OA patients’ intervention plan. So, to accomplish it, they have to rely on the information gathered in the evidence, do a proper patients’ clinical signs and symptoms evaluation, integrate the patient’s preferences, collect the patients’ socioeconomic status information and co-morbidities, listen to the patient’s main issues and goals, and delimit by their clinical experience what are the most mastered interventions (Quotations 58 and 59). However, it was also not uncommon to find that many of them feel more limited in their practice because they have to follow the physiatrist’s intervention plan prescription (Quotation 60 and 61).
*Quotation 58: “I always try to design the intervention plan between the PT and the patient. Of course, most of the treatments come from the PT, but I always try (more and more) from dialogue, to create goals that are important to the patient and, from there, the intervention is built, of course according to what science says.” [FT_1]*
*Quotation 59: “It all depends on the case-by-case assessment. But, we tend to make an assessment … especially if we are talking about OA, we are talking about a situation that has been going on for some time, a progressive situation. Therefore, what I normally do is, from the outset, the assessment always considers the exclusion of red flags, i. e., other things besides those of OA, which maybe contraindications or need more care or a more in-depth assessment. From there, I try to understand within what ... is chronic pain and the pain of the patient, and try to understand to what extent of all is formed. I. e., what slice size does the tissue injury component itself have, what slice size does the component of other factors that are usually not considered (issues such as sleep, fear of movement, physical inactivity). Make this assessment more global, to understand the extent on which I will have to act in each field. From that moment on, direct the strategies, depending on the assessment that’s being made.” [FT_7]*
*Quotation 60: “... patients go the physiatrist’s consultation before initiating physical therapy ...” [FT_3]*
*Quotation 61: “Usually it’s the physiatrist who sends a clinical indication of what are techniques that needs to be used and normally, as a PT, I try to adapt the techniques (I have the ability to select them for the situation, obviously discussed with the patient what we can do). But normally we don’t deviate much from the clinical indication and change only the techniques that are within our reach (the most functional techniques). We normally maintain, electrical therapy techniques, or physical agents – if they are appropriate – the ones that are prescribed.” [FT_4]*
Regardless of how the intervention plans are designed, to combine and order the various interventions during the treatment session, the rationale will depend on the effects that the PT defines for each intervention. Usually, to order the treatment plan interventions the previous intervention, in addition to have a specific effect, will also help in carrying out the succeeding interventions. For example, a PT referred that in addition to the effect of reducing stiffness and muscle tension (which itself can be beneficial for the patient), the use of Heat as a first intervention can facilitate the performance of other techniques, such as Massage and afterwards Exercises (Quotation 62).
*Quotation 62: “… what we usually do is, first, a component of tissue muscle relaxation (we often use some kind of heat, which helps with stiffness, helps to reduce muscle tension, and facilitates the work that we do afterwards), then we use manual therapy (massage, physiological and accessory mobilizations), and exercise or balance training and gait training to patients that need it.” [FT_3]*
Interventions are applied (more often) 2 to 3 times per week (Quotation 63). However, there are still patients who are treated daily (Quotation 64). The number of sessions applied depends on the workplace and the treatment session “recovery” (patients’ signs and symptoms easing) (Quotations 65 and 66). Nevertheless, with the patients’ clinical state evolving positively, treatments sessions could be once per week or twice per month, until patients are discharged and be self-sufficient in managing their difficulties (Quotation 67).
*Quotation 63: “Q: How many sessions do you do per week? 2 or 3 sessions” [FT_10]*
*Quotation 64: “Q: And those knee OA patients, how many sessions they do per week? At the unit, they do it every day.” [FT_6]*
*Quotation 65: “Q: Taking in consideration these patients, how many sessions do they do per week? I only go to the clinic twice a week, so maximum twice and minimum once.” [FT_5]*
*Quotation 66: “Q: How many sessions do they normally have at the clinic?*
*Usually twice a week.*
*Q: Why twice a week? Who define that number?*
*Usually, it’s us [PTs], and in our evaluation. In particular, they do it on average twice a week, because it’s the period that we saw that they were able to recover. For example, the day after the session they are a little more ‘slaughtered’, with the exercises and everything, and then spacing 2 or 3 days each intervention, it was the time that we saw that they were able to have more benefits.*
*Q: For example, do it on a Monday and a Thursday.*
*Or Tuesday and Friday, yes.*
*Q: Ok. Give at least 48–72 hours of rest. Is that it?*
*Exactly. Which I think, (I’m not sure) what most articles say. That OA treatment should be two to three times a week...” [FT_9]*
*Quotation 67: “Q: How many sessions they do per week?*
*This is usually defined at the beginning ... I already had patients doing two to three times per week, as patients doing two sessions each two weeks, and then going on only weekly or twice per month.*
*Q: For some particular reason?*
*For me it has a lot to do with the question that the patient has completed the exercise, or is able to make a plan at home or not. Sometimes there are patients who are working and we try to see what the patient’s schedule. If the patient isn’t very available for physical therapy, what I try to do is trying to talk to the patient and as long as he/she stick to the established plan, at home, it’s perfectly plausible to have weekly sessions.” [FT_8]*
As showed, the patient’s signs and symptoms have a very important role in both treatment plan interventions selection, and the treatment sessions per week decision and progression. There are several signs and symptoms related with OA stated by the PTs (Quotations 68, 69 and 70). The most important OA sign and symptom was knee pain, followed by activities of daily living limitations, lower limb muscular weakness and knee ROM limitations. By far, knee pain was the most important OA sign and symptom (referred 58 times in the PTs discourses) (Quotation 71). Although PTs believe that many of the signs and symptoms are related to pain, they also point out that most of the times, in a superficial analysis, pain may seem as the main patient problem, but when doing a deeper analysis to the patient’s speech at the anamnesis, his/her real problem is not being able to perform his/her activities of daily living (Quotations 72, 73, and 74). Therefore, while they still consider pain as the main sign and symptom, the importance given to activities of daily living limitations should be reviewed.
*Quotation 68: “Q: For you, and according to your experience, what are the most important signs and symptoms in knee OA?*
*The first is pain, which is why people come to us. And then joint stiffness, decreased strength and eventually knee ROM limitation. There are also other symptoms that are frequent, such as knee crepitus. Although, I personally don’t value it so much and don’t put it in the same level of pain, stiffness or decreased strength.” [FT_1]*
*Quotation 69: “Q: And for you, what are the main signs and symptoms of the OA population?*
*Pain, joint limitation, sensibility changes, muscular weakness, instability ...” [FT_5]*
*Quotation 70: “Q: And what about the patients who sought physical therapy?*
*In terms of clinic, for pain.*
*Q: Pain. Any other important sign or symptom?*
*It’s mostly pain. Patients, I think, only remember to do treatments for pain, not so much for prevention. At least here.*
*Q: And the other way around, for you, what are the main signs and symptoms of the OA population?*
*It’s limp, pain, inflammation … that’s what many of them have when they come to me, and limitations.*
*Q: Limitations? You’re talking about joint limitations, correct?*
*Yes.*
*Q: Here when you talk about limitation, can we also include some activities of daily living limitations?*
*Yes, consequently they couldn’t do it afterwards, especially women, ... it’s dramatic because they couldn’t do many activities.” [FT_10]*
*Quotation 71: “Q: According to the patients who arrived with knee OA, what are the most important signs and symptoms that they referred?*
*Pain ... mostly pain.” [FT_8]*
*Quotation 72: “Q: Try to remember the signs and symptoms that are more common in this type of pathology. What are the reasons for people seek physical therapy?*
*It’s like that, I would tend to respond directly, pain. Although, I consider that it’s not quite true. Because when we go to talk to the patient and explore a little bit what he/she wants, it’s to make their lives more independently and with a greater capacity. In other words, what normally leads them to look for it, I believe, it’s the question of pain, it’s the sign. Because this is actually something that then limits them. But … I saw a study some time ago (but then I lost it), which was almost a ranking of problems. I. e., what were the outcomes that the patient was expecting, and what were the outcomes that the PTs consider important ... and the thing didn’t match well. Normally we are always concerned with ROM and with pain, and patients want functionality and disability reduction. So, generally, I would tend to say that the main and most direct cause is pain, and then people report something more than pain: ‘I stopped being able to walk my dog’, or ‘I stopped being able to pick up my grandson or play with him/her on the floor because I can’t get up’. When we go more in detail, the concern is more this, i. e., the inability to perform activities of daily living.” [FT_7]*
*Quotation 73: “Q: And what are the reasons why they seek physical therapy?*
*I work in a village, in a very small area, and have a lot of rural population. And, above all, it’s to maintain functionality. They come to us when they have difficulty shopping, going to the garden or doing other activities of daily living. So, it’s mainly for maintenance and improvement of functionality.*
*Q: Any other secondary reason for seeking physical therapy?*
*Yes. To also relieve pain.*
*Q: Functionality and pain, with functionality first and pain second ...*
*Yes, exactly.” [FT_9]*
*Quotation 74: “Q: And what activities did they missed the most?*
*So, for men it was more not being able to go to the farm. Because I live in a country place and they couldn’t go because they had a lot of pain … women, it was more domestic chores.” [FT_10]*
As a form of summary, the PTs found it essential to reinforce that: (1) physical therapy interventions are vital to manage knee OA patients; (2) there are still few PTs in the primary health care, which can be fundamental in managing knee OA progression; and, (3) it is necessary to change the national health system, giving to the PTs more competence and autonomy (Quotation 75).
*Quotation 75: “... I think things are still very badly set up. Because primary health care at the moment isn’t providing answers. There are almost no PTs in primary health care. People continue to think that surgery is completely unavoidable.*
*(…)*
*Q: But don’t you think that this is a little bit influenced by how the system is currently set up? I. e., before a patient start doing physical therapy, he/she usually goes to a doctor, and the doctor doesn’t think that physical therapy can help in such cases…*
*Yes. That’s why I also think that updating doctors would be very important. We should be the first contact in health centers. (…) Effectively, knee OA care should all be done in primary care, with exercise, with educating people (explaining what they should do to prevent disease progression or, at least, to maintain functionality). In fact, surgery is an option, but only as a last option, and when other interventions have failed. Things are very badly set up.” [FT_8]*

**Suppl. Table 7.** Informed Consent Statement - Questionnaire.

**Informed Consent Statement**
**Daily Practice in Knee Osteoarthritis**
(Study title)
First of all, we appreciate your interest in participating in our questionnaire.
This questionnaire is being carried out at Faculdade de Desporto da Universidade do Porto, as part of a doctoral dissertation, in the Physical Therapy area.
The aim of this study is to characterize the Physical Therapists working class in Portugal and to know which are the most common interventions used in knee osteoarthritis patients.
For that, we are looking for Physical Therapists who: can read and write Portuguese; have completed in Portugal the enter-level degree for working as Physical Therapists; work or worked recently (in the last 6 months) as Physical Therapists in Portugal; have treated a knee osteoarthritis patient in the last 6 months.
During the questionnaire there will be no right or wrong answers, we are simply interested in your options and opinions on knee osteoarthritis. Furthermore, throughout the questionnaire, your participation will be anonymous and the data collected will be confidential, non-transferable, only used statistically in the academic context.
If you think that you will fit the population type and that you agree with the data treatment method, we would be very grateful for your participation in our questionnaire. Your feedback would be very important to us!
1. I declare that I have been informed of the questionnaire objectives and that I have been guaranteed that all participants identification data are confidential and that anonymity will be maintained. I know that I can refuse or interrupt participation in the study at any time, without any type of penalty. I understood the information that was given to me, I had the opportunity to ask questions and my doubts were clarified. In this way, I also authorize the results obtained to be reveled in the scientific community, guaranteeing confidentiality and anonymity. I agree to participate freely in the study mentioned above □ I do not agree to participate in the study mentioned above □

**Suppl. Table 8.** Semi-Structured Interviews script.

1. Are you familiar with patients with ***knee osteoarthritis***?
1.1. How many patients with this type of condition do you usually treat in a week?
1.2. How many treatment sessions do you usually perform per week for this type of patients?
1.3. For you, what are the most important signs and symptoms in this type of population?
1.4. Taking these patients into consideration, what are the most common interventions in your daily work?
1.4.1. What are the reasons for using/Why do you usually choose/How do you use these interventions?
1.4.2. What other treatment options have you already used?
1.4.2.1. Why did you stop using them?
1.4.3. Would you have an “open mind” to use other, more recent or lesser known interventions, for these cases?
1.4.3.1. If so, which is the reason?
1.4.3.2. If not, why?

**Suppl. Table 9.**
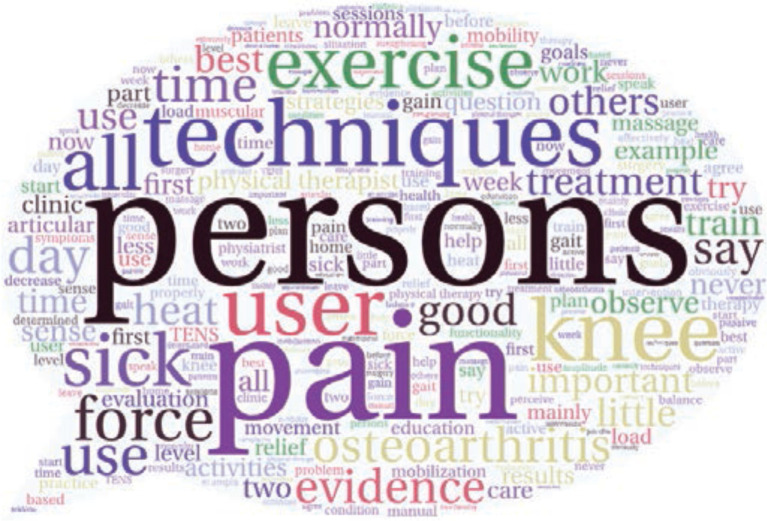
Word cloud.

## ABBREVIATIONS

CPGs: Clinical practice guidelines
EBP: Evidence-based practice
IFC: Interferential current
NMES: Neuromuscular electrical stimulation
OA: Osteoarthritis
PT: Physical therapist
ROM: Range of motion
TENS: Transcutaneous electrical nerve stimulation
UK: United Kingdom
US: Ultrasound therapy
